# Infantile Scurvy and "Sterilised" Milk

**Published:** 1902-03-01

**Authors:** 


### INFANTILE SCURVY AND "STERILISED" MILK.

The perennial controversy as to whether or not milk should be sterilised has received a very interesting contribution from the pen of Dr. Ransom,[1] of Nottingham, who, referring to the alleged possibility of scurvy arising from the prolonged use of boiled milk makes the important suggestion that it is not the sterilisation, but the imperfect sterilisation and consequent partial decomposition of the milk, which is the cause of the mischief. He refers to the observations made by Mr. F. A. Jackson and Dr. Vaughan Harley a couple of years ago, on the etiology of scurvy. This disease has been generally held to be due to the absence in the diet of some constituent of fresh food, and especially of fresh vegetable food, and it is generally believed that the administration of fresh vegetables or lime juice in addition to meat and preserved foods will prevent it. These observers, however, showed that in some cases a regular allowance of limejuice had failed to prevent scurvy, and that often, as witness the celebrated case of Dr. Nansen, no scurvy arose even when for months together nothing but freshly killed meat was eaten without any vegetables at all, and they came to the conclusion that the cause of scurvy is not the deprivation of some hypothetical substance contained in fresh food, but an actual poisoning by the ptomaines of tainted animal food. They brought the matter to the proof and showed that tainted animal food did, in fact, produce scorbutic symptoms in monkeys even while these animals were taking plenty of fresh vegetables. With this in his mind, Dr. Ransom comes to the question of infantile scurvy. He says : "All the cases of infantile scurvy that I have seen have been due to the sole use of proprietary dried foods. However perfect the food may appear to be from the point of view of chemical composition, there is no doubt that their prolonged use (for example, for six to eight months) does, in a certain number of cases, cause scurvy, although the child may for a time have gained weight and appeared thriving." Sir Thomas Barlow, however, in a letter to Dr. Ransom, goes further. He says, "undoubtedly the proprietary foods are the worst offenders ; condensed milk is responsible for a fair number of cases, and sterilised and humanised milk as supplied by the dairies is, if used long enough, very apt to produce scurvy. By this preparation, I mean milk which has been deprived of half its quantity of casein and has been subsequently sterilised, and in several cases stored for some weeks." He adds, "I have had several cases of scurvy in children taking milk sterilised by Soxhlet's process, which takes 40 to 45 minutes. I have not seen scurvy in children brought up with milk just scalded."

Dr. Ransom then points out that when so-called "sterilised" milk has been brought from a dairy, laboratory, or manufactured at home, there is a tendency to undue confidence in the virtues of sterilisation and to get a fresh supply at too long intervals. "Milk sent down from a London 'laboratory' is frequently given when a week old. But it is by no means true that milk which has been boiled, even for three-quarters of an hour, can be absolutely relied on as sterile. It happens not very rarely that 'sterilised' milk from the best London dairies is found when the bottle is opened to be obviously bad to smell and taste, and it is practically certain that minor fermentive changes may go on in milk which seems good enough to give to the child. Hence we may be for several months administering to the infant small doses of the chemical products of saprophytic or pathogenic germs." Dr. Ransom holds then that the evil effects observed not rarely after the prolonged use of powdered substitutes for milk, and occasionally after the use of modified milk supposed to be thoroughly sterilised, may be partly due to unsuitable chemical composition as to food elements, but more largely to actual insufficient sterilisation, and subsequent keeping the food long enough so allow decomposition processes to occur. The moral then of all this is that when milk has been boiled it should be used. There is, says Dr. Ransom, no solid evidence to show that milk raised to its boiling point or to the temperature of boiling water for 10 minutes or a quarter of an hour suffers any diminution of its nutrient qualities. Neither is it probable that if consumed within 24 hours of the heating it will cause infantile scurvy.

[1] Brit. Med. Jour., Feb. 22.

